# Identification of Triple-Negative Breast Cancer Genes and a Novel High-Risk Breast Cancer Prediction Model Development Based on PPI Data and Support Vector Machines

**DOI:** 10.3389/fgene.2019.00180

**Published:** 2019-03-15

**Authors:** Ming Li, Yu Guo, Yuan-Ming Feng, Ning Zhang

**Affiliations:** ^1^Department of Biomedical Engineering, Tianjin Key Lab of BME Measurement, Tianjin University, Tianjin, China; ^2^Department of Radiation Oncology, Tianjin Medical University Cancer Institute and Hospital, Tianjin, China

**Keywords:** triple-negative breast cancer, gene, proteins, protein-protein interaction network, SVM

## Abstract

Triple-negative breast cancer (TNBC) is a special subtype of breast cancer that is difficult to treat. It is crucial to identify breast cancer-related genes that could provide new biomarkers for breast cancer diagnosis and potential treatment goals. In the development of our new high-risk breast cancer prediction model, seven raw gene expression datasets from the NCBI gene expression omnibus (GEO) database (GSE31519, GSE9574, GSE20194, GSE20271, GSE32646, GSE45255, and GSE15852) were used. Using the maximum relevance minimum redundancy (mRMR) method, we selected significant genes. Then, we mapped transcripts of the genes on the protein-protein interaction (PPI) network from the Search Tool for the Retrieval of Interacting Genes (STRING) database, as well as traced the shortest path between each pair of proteins. Genes with higher betweenness values were selected from the shortest path proteins. In order to ensure validity and precision, a permutation test was performed. We randomly selected 248 proteins from the PPI network for shortest path tracing and repeated the procedure 100 times. We also removed genes that appeared more frequently in randomized results. As a result, 54 genes were selected as potential TNBC-related genes. Using 14 out the 54 genes, which are potential TNBC associated genes, as input features into a support vector machine (SVM), a novel model was trained to predict high-risk breast cancer. The prediction accuracy of normal tissues and TNBC tissues reached 95.394%, and the predictions of Stage II and Stage III TNBC reached 86.598%, indicating that such genes play important roles in distinguishing breast cancers, and that the method could be promising in practical use. According to reports, some of the 54 genes we identified from the PPI network are associated with breast cancer in the literature. Several other genes have not yet been reported but have functional resemblance with known cancer genes. These may be novel breast cancer-related genes and need further experimental validation. Gene ontology (GO) enrichment and Kyoto Encyclopedia of Genes and Genomes (KEGG) enrichment analyses were performed to appraise the 54 genes. It was indicated that cellular response to organic cyclic compounds has an influence in breast cancer, and most genes may be related with viral carcinogenesis.

## Introduction

Breast cancer is a malignant tumor that is highly prevalent among women worldwide. In recent years, the incidence rate has increased significantly. According to estrogen receptor (ER), progesterone receptor (PR), and human epidermal growth factor receptor 2 (HER-2) status, breast cancer can be classified into four categories. Triple-negative breast cancer (TNBC), one of the more specialized types of breast cancer, is defined as the lack of expression of the ER and PR, as well as breast cancer that lacks HER-2 overexpression or gene amplification. TNBC is more common in young women, with large tumors, high lymphatic metastasis rate, and high clinical stage. The 5-year recurrence rate is high, and visceral metastases such as liver and lung metastasis are more common. Compared with other types of breast cancer, TNBC has characteristics of rapid tumor growth, early recurrence, easy metastasis, and so on (Prat et al., 2013). Up to now, the genes related to this disease are poorly understood.

Triple-negative breast cancer accounts for about 15–25% of all breast cancers. The identification of disease-related genes and prediction of high-risk breast cancer patients have become important problems. Genes that are highly associated with TNBC can be found using gene expression profiles. However, there are still some problems in the current methods of predicting protein function using high-throughput protein interaction data. It usually has a high false positive rate, and the reliability of functional prediction results is reduced (Li et al., 2012b; Oliver et al., 2015).

In recent years, the continuous accumulation of protein interaction data has made it possible to analyze and predict protein functions at the system level through the protein-protein interaction (PPI) network. Nabieva et al. (2005) proposed the “guilt-by-association rule” (GBA), which states that interacting proteins have the same or similar functions, which suggests that protein function can be predicted by protein interactions.

In this study, we identified TNBC-related genes by a computational method. A weighted functional PPI network was integrated, which can overcome the disadvantages of that by only using the gene expression profiles. We also previously successfully applied such an integrating method to gene function prediction and to the identification of novel genes of various kinds of diseases, such as influenza A/H7N9 virus infection (Ning et al., 2014), colorectal cancer (Li et al., 2012b), lung cancer (Li et al., 2013b), colorectal cancer (Li et al., 2013a), hepatitis B virus (HBV) infection-related hepatocellular carcinoma (Jiang et al., 2013), retinoblastoma (Li et al., 2012c), Ebola virus (Cao et al., 2017), etc.

## Materials and Methods

**FIGURE 1:**
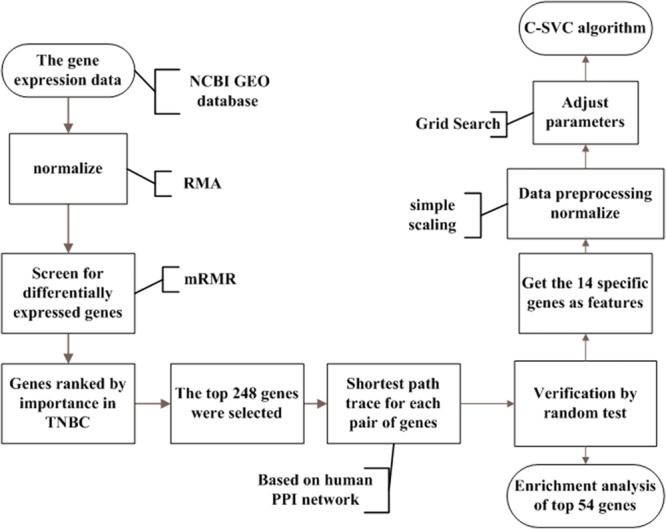
The analysis flowchart for this study. This method integrated breast cancer gene expression data and PPI data. Firstly, we regard each gene as a feature in the data and used mRMR to rank the importance of the genes. Then we selected the top 248 genes from the mRMR results. We searched the shortest paths between every pair of the 248 coding proteins by the Dijkstra algorithm in the PPI network. Shortest path proteins were retrieved and were ranked in descending order. After that, 54 of the shortest path proteins were selected and were considered as the potential triple-negative breast cancer-related genes. Finally, using the C-SVC model for classification in order to achieve satisfactory results, we used the grid Search method to select the appropriate parameters.

The whole process of our study is illustrated in Figure 1. Details are presented in the following sub-sections.

### Dataset

Expression profiles from datasets GSE31519, GSE9574, GSE20194, GSE20271, GSE45255, and GSE15852 were obtained from the GEO database^1^. The dataset involves 319 sample chips with 101 normal breast tissue samples and 218 TNBC tissue samples (including 21 Stage II samples and 101 Stage III samples).

In this study, the robust multi-array average (RMA) method in “limma” in R was used to normalize microarray data and to perform a log_2_ transformation of chip data. In total, 12,437 genes were obtained. RMA uses a multi-chip model that requires standardization of all chips together. The expression value is estimated based on a stochastic model employed by the perfect match (PM) signal distribution. It is currently the most common chip data preprocessing method. RMA is commonly used in the literature. This method has also been used in many other biomedical research problems, such as when analyzing diabetic nephropathy (Cohen et al., 2008), the crosstalk between B16 melanoma cells and B-1 lymphocytes (Xander et al., 2013), colon cancer (Melo et al., 2013), etc.

### The mRMR Method

We employed the mRMR method (Peng et al., 2005; Li et al., 2012a,b; Zhang et al., 2012; Zou et al., 2016b; Su et al., 2018) to rank the importance of all 12,437 genes examined. In such a procedure, each gene was regarded as a feature. The Maximum Relevance criterion selects features most important in discriminating TNBC samples and controls. The Minimum Redundancy criterion excludes redundant features among the selected ones. In an mRMR procedure, a value A-B is calculated for each feature, in which value A is represented for the relevance and value B for the redundancy of the feature. Then the features are ranked by their A-B values in descending order to reflect the importance to the target. The most important feature is ranked at the top (Peng et al., 2005; Li et al., 2012a,b; Zhang et al., 2012; Zou et al., 2016b).

Two ordered lists were generated by the mRMR method, one was called the MaxRel table, and the other was called the mRMR table. In the MaxRel table, all the features were ranked only by the Maximum Relevance criterion. In the mRMR table, they were ranked by the mRMR criterion, i.e., a feature with a smaller index in such a table could be more important since it has a better trade-off between the maximum relevance and the minimum redundancy. In this study, we selected the top 248 features from the mRMR table, with which the corresponding 248 genes were regarded as significantly differentially expressed genes from the expression profiles and were analyzed in the downstream procedures.

### PPI Network From STRING

The STRING database (version 10.0)^2^ (Franceschini et al., 2013) is a database for searching for known and predicted interactions between proteins. The related interactions mentioned herein include direct and indirect relationships between proteins. The interacting protein can be mapped to a weight network in STRING. In such a network, proteins are denoted as nodes and the interaction of every two proteins is given as an edge marked with a confidence score. If the confidence score is higher, they may have more analogous functions (Kourmpetis et al., 2010; Ng et al., 2010; Szklarczyk et al., 2011). In this study, we used a *d* value instead of a confidence score (s) for the weight of each interaction edge. According to the equation *d* = 1,000-*s, d* was calculated. Therefore, the *d* value can be considered to represent the protein distances to each other; a smaller distance value indicates the protein pair has a higher interaction confidence score.

In this study, the human PPI data in the STRING database were selected as the data source, and there are 8,548,002 pairs of related interaction forces. The ID of the human species is 9,606.

### Shortest Path Tracing

Interactions between every protein pair were analyzed in a graph. In this study, the R package “STRINGdb” was used to map the corresponding protein IDs of the top 248 genes selected by mRMR. The betweenness of a shortest path protein is the number of shortest paths across the protein. Then, the shortest path proteins were ranked by betweenness in descending order. The proteins whose betweenness was greater than 3,000 were picked out and their corresponding genes were treated as breast cancer-related genes. The Dijkstra algorithm served to find the shortest path in the graph *G* between two given proteins, which was implemented in the R package “igraph” (Csardi and Nepusz, 2006). In order to ensure the validity and precision of our results, we randomly chose 248 proteins in the PPI network for shortest path tracing and repeated the procedure 100 times, and a permutation test was performed. Then we removed 5 genes that appear more frequently in randomized results.

### The C-SVC Algorithm

The support vector machine (SVM) method largely overcomes the dimensional disaster and local minimization of feature attributes in traditional machine learning and solves small samples. There are many advantages in non-linear and high-dimensional pattern recognition, which have received more and more attention in the fields of biomedicine and bioinformatics. Therefore, in the field of health care, an improved SVM algorithm for the diagnosis of breast cancer diseases was applied by Zhang et al. (2013). A new data feature dimension reduction method for lymphatic diseases was proposed by Azar et al. (2014). Auxiliary diagnosis has achieved a certain improvement in diagnostic efficiency (Yuan et al., 2010; Mokeddem et al., 2013).

The Cost Support Vector Classification (C-SVC) is a method of SVM classification. It introduces penalty parameter C for SVM classification.

(1) $$\begin{array}{c} \min_{w,b,\zeta }\frac{1}{2}w^{T}w+C\sum_{i=1}^{n}\zeta_{i} \end{array}$$

subject to $y_{i}(w^{T}\;\phi (x_{i})+b)\geq 1-\zeta_{i}$

$$\begin{array}{c} \zeta_{i}\geq 0,\;i=1,\ldots ,n \end{array}$$

Its dual is:

(2) minα12 αTQα−eTα

subject to y^T^α = 0

$$\begin{array}{c} 0\leq \alpha_{i}\leq C,\;i=1,\ldots ,n, \end{array}$$

where *e* is the vector of all ones, C > 0 is the upper bound, *Q* is an *n* by positive semidefinite matrix, $Q_{ij}\equiv y_{i}y_{j}K\left(x_{i},x_{j}\right)$, where $K\left(x_{i},x_{j}\right)=\phi {\left(x_{i}\right)}^{T}\phi \left(x_{j}\right)$ is the kernel. Here, training vectors are implicitly mapped into a higher (maybe infinite) dimensional space by the function:

(3) sgn(∑i=1nyi αiK(xi,xj)+ρ)

The C-SVC is capable of categorizing two types of breast tissue (Jiang and Yao, 2016).

### Data Preprocessing for the Prediction Model

To test the accuracy of the C-SVC-based high-risk breast cancer prediction model, we divided the samples into two groups, one for normal tissue and breast cancer tissue, and the other for Stage II and Stage III breast cancers.

Scaling data according to the Equation (4):

(4) $$\begin{array}{c} y'=lower+\left(upper-lower\right)\;*\;\frac{y-min}{max-min} \end{array}$$

where *y* is the data before scaling, y′ is the scaled data; *lower* is the lower bound of the data specified in the parameter, *upper* is the upper bound of the data specified in the parameter; *min* is the minimum of all training data, and *max* is the maximum value of all training data.

The preprocessing of the data has a great influence on the final classification accuracy. This paper will compare the different preprocessing methods and finally choose the method with high classification accuracy to establish the model.

### Parameter Optimization

The choice of a kernel function is important. In a specific problem, several kernel functions should be applied in order to choose the best one, obtaining the highest accuracy (Deng et al., 2016). Both the type of kernel function and other parameters such as penalty parameter C and γ in kernel functions impact the performance. Thus, we use the grid search method to select the appropriate parameters.

## Results

### The Top 54 Genes on PPI Shortest Paths

**FIGURE 2:**
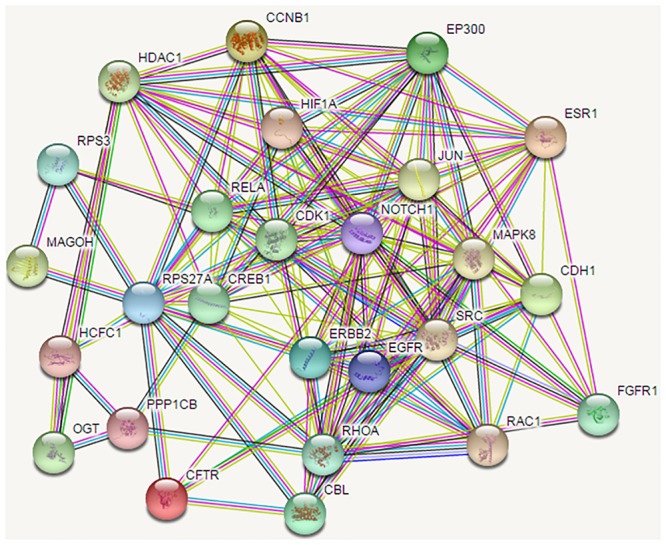
The protein-protein interaction network of the proteins encoded by the 54 candidate genes. Shortest path proteins were retrieved from the shortest paths between every protein pair coded by the top 248 genes selected from the mRMR table. The shortest path between every protein pair was searched by the Dijkstra algorithm in the network. Finally, the 54 shortest path proteins were obtained, the related genes of which were considered as candidate genes. The PPI network of the 54 shortest path proteins is depicted, in which the nodes represent proteins, and the lines between nodes represent protein interactions.

After removing the five randomized genes from the intersection of the shortest path results for normal breast and TNBC tissues, a total of 54 genes associated with TNBC were obtained, as shown in Table 1. Similarly, we mapped the PPI networks of these 54 genes using the STRINGdb package in R, as shown in Figure 2.

**Table 1:** The 54 candidate breast cancer-related genes and betweenness.

hgnc_symbol	ensp	Betweenness	Reference
*MAGOH*	ENSP00000360525	1777	Kataoka et al., 2001
*CBL*	ENSP00000264033	1548	Thien and Langdon, 2001
*RPS3*	ENSP00000433821	1380	Jang et al., 2004
*FGFR1*	ENSP00000393312	1286	Pirvola et al., 2002
*RHOA*	ENSP00000400175	1237	Strutt et al., 1997
*EP300*	ENSP00000263253	1048	Gayther et al., 2000
*RAC1*	ENSP00000348461	871	Gu et al., 2003
*CDK1*	ENSP00000378699	852	Santamaría et al., 2007
*CDH1*	ENSP00000261769	848	Konishi et al., 2004
*EGFR*	ENSP00000275493	815	Kobayashi et al., 2005
*JUN*	ENSP00000360266	811	Curran and Franza, 1988
*NOTCH1*	ENSP00000277541	803	Nicolas et al., 2003
*HCFC1*	ENSP00000309555	795	Jolly et al., 2015
*OGT*	ENSP00000362824	786	Sodi et al., 2015
*PPP1CB*	ENSP00000296122	786	Alquobaili et al., 2009
*CFTR*	ENSP00000003084	767	Zhang et al., 2013
*ERBB2*	ENSP00000269571	763	Blackwell et al., 2010
*HIF1A*	ENSP00000338018	762	Ponente et al., 2017
*ESR1*	ENSP00000206249	745	Robinson et al., 2013
*HDAC1*	ENSP00000362649	705	Kawai et al., 2003
*RPS27A*	ENSP00000272317	672	Wang et al., 2014
*RELA*	ENSP00000384273	658	Xia et al., 2010
*CREB1*	ENSP00000387699	582	Chhabra et al., 2007
*CCNB1*	ENSP00000256442	565	Ding et al., 2014
*MAPK8*	ENSP00000353483	561	Slattery et al., 2015
*SRC*	ENSP00000350941	559	Zhang et al., 2013
*OPTN*	ENSP00000263036	558	Jeffrey et al., 1999
*ITGB1*	ENSP00000303351	553	Yang et al., 2016
*RPS2*	ENSP00000341885	549	Douglas, 1997
*NFKB1*	ENSP00000226574	512	Curran et al., 2002
*MT-ATP6*	ENSP00000354632	508	You et al., 2009
*MT-CO3*	ENSP00000354982	508	
*ATP5A1*	ENSP00000282050	508	Lotz et al., 2014
*WDR5*	ENSP00000351446	466	Dai et al., 2015
*CREBBP*	ENSP00000262367	466	Gupta et al., 2014
*RAN*	ENSP00000376176	445	Yuen et al., 2013
*HNRNPK*	ENSP00000365439	429	
*BTRC*	ENSP00000359206	408	
*PXN*	ENSP00000228307	406	Sp et al., 2017
*CYC1*	ENSP00000317159	394	Han et al., 2016
*CYCS*	ENSP00000307786	391	
*SHC1*	ENSP00000401303	383	Wagner et al., 2004
*MEF2A*	ENSP00000346389	381	
*NCOR2*	ENSP00000384018	362	Green et al., 2008
*LIN7A*	ENSP00000447488	347	Gruel et al., 2016
*PCNA*	ENSP00000368438	336	Kato et al., 2002
*YAP1*	ENSP00000282441	335	Yu et al., 2013
*MPP5*	ENSP00000261681	331	Van Rossum et al., 2006
*AMOT*	ENSP00000361027	331	Zhang and Fan, 2015
*RANGAP1*	ENSP00000348577	323	
*FOS*	ENSP00000306245	316	Langer et al., 2006
*STAT1*	ENSP00000354394	313	Khodarev et al., 2010
*AR*	ENSP00000363822	308	Subik et al., 2010
*SUMO2*	ENSP00000405965	297	Subramonian et al., 2014

### Function Gene Enrichment Analysis

**FIGURE 3:**
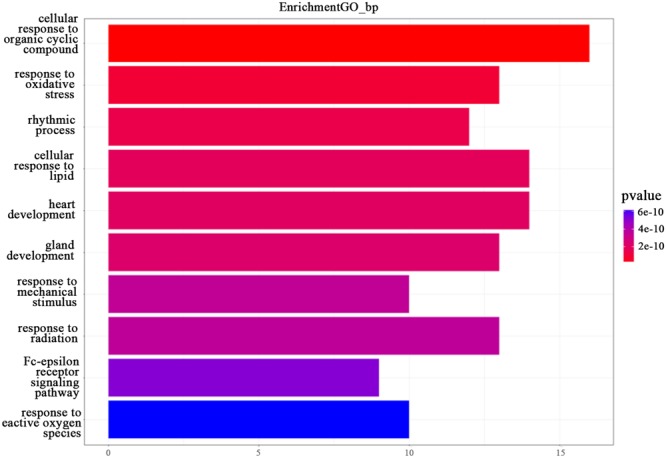
The GO enrichment analysis. The top 10 terms from the GO enrichment analysis ranked by *p*-value, shown as a bar chart. The GO terms by name are listed on the *y*-axis. The shared number of terms is shown as the length of histogram. The different colors represent the different *p*-values.

**FIGURE 4:**
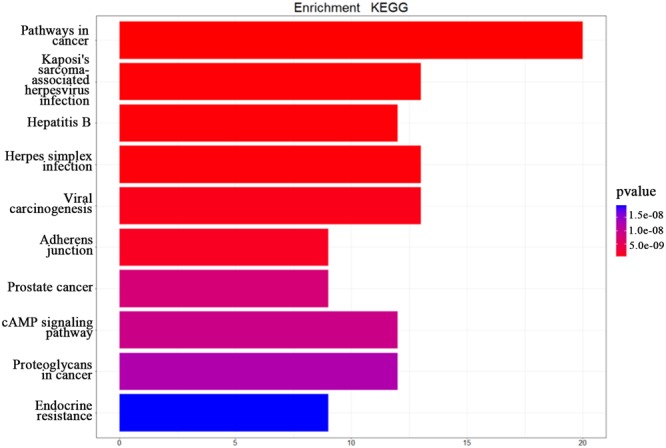
The KEGG enrichment analysis. The top 10 pathways from the KEGG enrichment analysis ranked by *p*-value, shown as a bar chart. The terms of the KEGG pathways are depicted on the *y*-axis. The shared number of pathways is shown as the length of histogram. The different colors represent the different *p*-values.

In this study, we transferred the disease-related genes into its corresponding EntrezID by using “org.Hs.eg.db” in R. Then, we analyzed the functional enrichment of the 54 candidate genes in KEGG pathways and GO terms using the R package “clusterProfilter.” The GO enrichment analysis includes three categories: cellular component (CC), molecular function (MF), and biological process (BP). In our study, we only focus on BP enrichment due to its importance. These terms were ranked by the enrichment *p*-value. The Benjamin multiple testing correction method was used to regulate family-wide false discovery rate under a certain rate (e.g., ≤0.01) to correct the enrichment *p*-value (Benjamini and Yekutieli, 2001). Results of the GO enrichment analysis ranked by *p*-value were provided in Table 2 and result of the KEGG enrichment analysis ranked by *p*-value was provided in Table 3, respectively. The top 10 terms of the enrichment results are depicted in Figure 3, 4.

**Table 2:** Results of the GO enrichment analysis.

Go term entry ID	Description	*p*-value	Count
GO:0071407	Cellular response to organic cyclic compound	1.217E-12	16
GO:0006979	Response to oxidative stress	9.108E-11	13
GO:0048511	Rhythmic process	1.467E-10	12
GO:0071396	Cellular response to lipid	1.897E-10	14
GO:0048732	Heart development	2.055E-10	14
GO:0009612	Gland development	2.349E-10	13
GO:0009314	Response to mechanical stimulus	3.549E-10	10
GO:0009314	Response to radiation	3.666E-10	13
GO:0038095	Fc-epsilon receptor signaling pathway	5.244E-10	9
GO:0000302	Response to reactive oxygen species	6.401E-10	10

**Table 3:** Results of the KEGG enrichment analysis.

KEGG term entry ID	Description	*p*-value	Count
hsa05200	Pathways in cancer	7.835E-13	20
hsa05167	Kaposi’s sarcoma-associated herpesvirus infection	1.406E-10	13
hsa05161	Hepatitis B	2.393E-10	12
hsa05168	Herpes simplex infection	3.262E-10	13
hsa05203	Viral carcinogenesis	9.155E-10	13
hsa04520	Adherens junction	1.430E-09	9
hsa05215	Prostate cancer	7.949E-09	9
hsa04024	cAMP signaling pathway	9.423E-09	12
hsa05205	Proteoglycans in cancer	1.249E-08	12
hsa01522	Endocrine resistance	1.915E-08	9

### High-Risk Breast Cancer Prediction

In this study, we implemented the C-SVC algorithm in the Matlab 2015a environment. The radial basis function (RBF) kernel function was employed in this study since the function has been widely used in various bioinformatics prediction problems and usually yields the best results compared to other types of kernel functions (Li et al., 2011; Song et al., 2011; Khan et al., 2016). In this study, we also employed other kernel functions on the same prediction task and found that the RBF performed the best (data not shown). The grid search method was used and the results were verified by the ten-fold cross-validation method. The data in the experiment was divided into 10 sets of similar size, and 9 of them were used in turn as the training set. One set was used as the test to calculate the corrections and errors of the prediction. As a result, the normal tissue and TNBC tissue prediction accuracy reached 95.394%, and the Stage II and Stage III TNBC predictions reached 86.598%, as shown in Table 4. It is indicated that based on the 54 genes as features, the C-SVC algorithm can accurately predict normal tissue and TNBC, as well as the stage data for TNBC.

**Table 4:** The performance of the high-risk breast cancer classification model.

	ACC	Precision	Recall	F-measure
Normal and TNBC	95.394%	88.889%	100%	94.118%
II and III	86.597%	80.952%	100%	89.474%

*The experiment result shows its upper recall and precision rate. Its recall rate reaches 100%. The precision and F-measure are also above 80%.*

## Discussion

### Genes Identified From PPI Shortest Paths

As can be seen from Table 1, some genes are associated with TNBC, such as *FGFR1, EGFR, NOTCH1, ERBB2, AR*, and so on.

Among these genes, *CBL, FGFR1, RHOA, EP300, RAC1, CDH1, EGFR, NOTCH1, ERBB2, HIF1A, HDAC1, CCNB1, SRC, ITGB1, NFKB1, CREBBP, PCNA, STAT*, and *AR* are reported to be related to TNBC.

#### The Migration and Invasion

We found that specific genes such as *CBL, RHOA, EP300, RAC1, CDK1*, and *CDH1* are involved in the migration and invasion of breast cancer.

*CBL* is a proto-oncogene, and it is indicated that *CBL* is associated with the development of leukemia. It has been found that this gene is mutated or translocated in many cancers (Choi et al., 2003). *CBL* encodes a protein which is one of the enzymes required to target substrate degradation through the proteasome. It has been found that the gene mutation or translocation occurs in many cancers, such as acute myeloid leukemia. So far, there are some studies suggesting that *CBL* is associated with breast cancer or TNBC. It is reported by Kales et al. that low expression of Cbl-c is associated with breast tumors (Kales et al., 2014). It is shown that this gene is involved in the invasion of cancer. The study by Crist et al. showed that a diminished regulatory capacity of Cbl-c is a recurrent event that may play a role in the invasive nature of colorectal cancer cells (Cristóbal et al., 2014). From these studies, it can be speculated that CBL is associated with invasiveness of TNBC.

In the Rho family, *RHOA* is a small GTPase protein. The overexpression of this gene is related to tumor cell proliferation and metastasis. It is shown that the RhoA pathway mediates the independent invasion of MMP-2 and MMP-9 in TNBS cell lines (Fagan-Solis et al., 2013). *RHOA* is the target of *miR-146a* to prevent cell invasion and metastasis in breast cancer (Liu et al., 2016). Lee et al. showed that *ODAM* expression maintains breast cancer cell adhesion and thus prevents breast cancer cell metastasis by modulating RhoA signaling in breast cancer cells (Lee et al., 2015). The study by Kwon et al. showed that *SMURF1* acts in EGF-induced migration and invasion of breast cancer cells (Kwon et al., 2013). In conclusion, *RHOA* is involved in the invasion of TNBC cells.

*EP300* (histone acetyltransferase p300) encodes the p300 transcriptional coactivator of the adenovirus E1A-associated cell. Studies by Cho et al. (2015) showed that p300 and *MRTF-A* synergistically enhance the expression of migration-associated genes in breast cancer cells. In addition, it is report that the EP300-G211S mutation correlates with a low mutation load in TNBC patients (Bemanian et al., 2017). Therefore, *EP300* is directly related to TNBC.

The *RAC1* gene encodes a protein belonging to the GTPase of the small GTP-binding protein RAS superfamily. It was found that *RASAL2* activates *RAC1* to promote TNBC (Feng et al., 2014). Studies by De et al. have shown that the caspase-β-catenin-RAC1 cascade suggests a link between *RAC1* and integrin-related metastasis in TNBC (De et al., 2017). In addition, studies by De et al. (2017) observed that two different mTORC2-dependent signaling pathways can be fused with *RAC1* to drive breast cancer metastasis. Therefore, *RAC1* may play an important clinical role for the treatment of TNBC.

*CDH1*, the gene encoding E-cadherin (E-cadherin), is a calcium-dependent cell adhesion protein belonging to the cadherin family. It is involved in the process of tumor proliferation, invasion, and metastasis. Therefore, it is anticipated that gene function defects will promote the occurrence and development of cancer. It is shown that 1α, 25-dihydroxyvitamin D3 induces E-cadherin expression in TNBC cells through demethylation of the *CDH1* promoter (Lopes et al., 2012).

#### Posttranscriptional Regulation of Gene Expression

We found that *FGR1, MAGOH, RPS3*, and *CDK1* are all involved in posttranscriptional regulation of gene expression.

*FGFR1* is one of the fibroblast growth factor (FGF) encoding genes. Cheng et al. suggested that upregulation of *FGFR1* expression in TNBC cells may be treated as a potential therapeutic target (Cheng et al., 2015). Vinayak et al. (2013) reported that FGF pathways have been implicated in breast tumorigenesis as a potential target for TNBC. In addition, there is some research indicating that it is related to breast cancer, as *FGFR1* was found to be associated with luminal A breast cancer (Zou et al., 2016a). FGFR is also helpful in the targeted therapy of breast cancer (Ye et al., 2014). Amplification of *FGFR1* also occurs in almost 10% of ER-positive breast cancers, particularly luminol type B breast cancer subtypes. In summary, *FGFR1* and TNBC are closely related.

*MAGOH* ranked first, indicating it plays an important role in TNBC. A protein encoded by the gene is the core component of the composite exon. There is some evidence showing that it is associated with TNBC. This gene could possibly be treated as a potential specific gene for TNBC.

The *RPS3* gene encodes the 40S ribosomal protein S3 domain. Kim et al. have shown that the rpS3 protein is a marker of malignancy (Kim et al., 2013). It is reported that it is mainly associated with lung cancer. Slizhikova et al. (2005) have shown that this gene is a marker of human squamous cell lung cancer.

*CDK1* is a set of Ser/Thr kinase systems corresponding to cell cycle progression. It was shown by Xia et al. (2014) that the *CDK1* inhibitor RO3306 potentiates BRCA-negative breast cancer cell responses to PARP inhibitors. *CDK1* inhibition may have a role in the adjuvant treatment of TNBC.

Additionally, some genes have also been reported to have a direct relationship with TNBC. In a nutshell, most of the specific genes found in this study have been reported to be associated with TNBC, while others are rarely reported to have a direct relationship with TNBC, suggesting that they could be new specific genes and potentially be new biomarkers for breast cancer prevention and treatment.

### Candidate Gene Enrichment Analysis

We used the ‘clusterProfilter’ package in R for the enrichment analysis of the 54 candidate genes, ranking the GO terms and KEGG pathways by *p*-value in ascending order. In the present study, the *p*-value was calculated for each KEGG and GO term.

In this study, we only focused on BP. The top 10 terms ranked by *p*-value are shown in Figure 2.

As shown in Figure 2, “cellular response to organic cyclic compound (GO:0071407)” was ranked first. It is well known that any process leading to changes in cell state or activity (changes in movement, secretion, enzyme production, gene expression, etc.) is the result of stimulation by organic cyclic compounds. It proved the importance of this BP in TNBC. Both “response to oxidative stress” (GO: 0006979) and “response to reactive oxygen species” (GO: 0000302) are related to the reaction of oxygen. “Rhythmic process” (GO:0048511), “cellular response to lipid” (GO:0071396), “heart development” (GO: 0007507), “gland development” (GO:0048732), and “glandular development” (GO:0048732) are also associated with TNBC. In addition, the two responses “response to mechanical stimulus” (GO: 0009612) and “response to radiation” (GO: 0009314) are also associated with TNBC, as well as the “Fc-epsilon receptor signaling pathway” (GO: 0038095). The above entry comment may provide some new ideas for TNBC.

The top 10 terms of KEGG enrichment ranked by *p*-value are depicted in Figure 3. It is clear that “pathways in cancer” (hsa05200) is ranked at the top, demonstrating its importance in TNBC.

In addition, “Kaposi’s sarcoma-associated herpesvirus infection” (hsa05167), “hepatitis B” (hsa05161), “herpes simplex infection” (hsa05168), and “viral carcinogenesis” (hsa05203) are associated with viral infection. Moreover, “adherens junction” (hsa04520), “prostate cancer” (hsa05215), “cAMP signaling pathway” (hsa04024), “proteoglycans in cancer” (hsa05205), and “endocrine resistance” (hsa01522) are also associated with the occurrence and development of TNBC. Huo et al. (2012) suggested that breast cancer and viral infection were statistically significant. From the enrichment analysis above it can be concluded that TNBC may be related to viral carcinogenesis.

### Advantages of the Method and Extension

It is anticipated that our model may become a useful tool for studying cancers from the angle of genes and networks. It was observed by analyzing the results that the specific genes, the biological functions of the significant genes, and the pathways enriched would contribute to cancer diagnosis and cancer predictions. Furthermore, the current model can also be used to solve many other disease prediction problems, and we also have many similar applications in our previous studies, such as for Ebola (Cao et al., 2017) and for A/H7N9 (Zhang et al., 2014). These studies show promising results and prove the efficiency of the proposed methods. However, this method has limitations on diseases with insignificant genes, which may lead to bias in prediction results. Additionally, insufficient samples will also affect the results. Moreover, genes identified from computational methods should be verified by further experimental studies.

In all, results may shed some light on the understanding of the mechanism of the tumorigenesis of breast cancer, providing new references for research into the disease and for the development of new strategies for clinical therapies as well as providing potential for future experimental validation.

## Conclusion

In this study, we developed a novel method to identify TNBC-related genes. This method integrated breast cancer gene expression data and PPI data. Many of the identified genes were reported to be related to TNBC in the literature. Most of these genes are related with invasion and metastasis. GO enrichment analysis indicated that the cellular response to organic cyclic compounds have an influence in breast cancer. KEGG pathway analysis indicated that most of these 54 genes may be related with viral carcinogenesis. We believe that these findings will provide some insights for breast cancer therapy and drug development.

We also developed a new SVM method based on the C-SVC for predicting high-risk breast cancer. The prediction accuracy of normal tissues and TNBC tissues reached 95.394%, and the predictions of Stage II and Stage III TNBC reached 86.598%.

Our method could be helpful for identifying novel cancer-related genes and assisting doctors in medical diagnosis. Identification of TNBC genes and a novel high-risk breast cancer prediction model development based on PPI data and SVM method may have certain theoretical significance and practical value in the application of cancer diagnosis. Recently, link prediction paradigms have been applied in the prediction of disease genes (Zeng et al., 2017a,b), circular RNAs (Zeng et al., 2017c), and miRNAs (Liu et al., 2016). Additionally, computational intelligence such as neural networks (Cabarle et al., 2017) can be applied in this field.

## Data Availability

The datasets generated for this study can be found in NCBI GEO, GSE31519, GSE9574, GSE20194, GSE20271, GSE32646, GSE45255, and GSE15852.

## Author Contributions

NZ conceived and supervised the project. YG and ML were responsible for the design, data preprocessing, computational analyses, and drafted the manuscript with revisions provided. Y-MF, NZ, and ML participated in the design of the study and performed the computational analysis. All authors read and approved the final manuscript.

## Conflict of Interest Statement

The authors declare that the research was conducted in the absence of any commercial or financial relationships that could be construed as a potential conflict of interest.

## Footnotes

1 http://www.ncbi.nlm.nih.gov/geo/

2 http://string.embl.de/
